# Supporting Students with Disabilities during the COVID-19 Pandemic: The Perspective of Disability Resource Professionals

**DOI:** 10.3390/ijerph20054297

**Published:** 2023-02-28

**Authors:** Katherine C. Aquino, Sally Scott

**Affiliations:** 1 Department of Administrative and Instructional Leadership, St. John’s University, Queens, NY 11439, USA; 2 Association on Higher Education and Disability, Huntersville, NC 28070, USA

**Keywords:** students with disabilities, higher education, student support, disability support services, administrators

## Abstract

Utilizing national survey data, this paper details the academic and access challenges created by the pandemic for students with disabilities, as perceived by disability resource professionals. Data included in this paper capture disability support service challenges at two unique timepoints during the COVID-19 pandemic—May 2020 [*n* = 535] and January 2021 [*n* = 631]. Disability resource professionals reported there was difficulty in the initial months of the pandemic for students to provide documentation of a disability to receive accommodations, use assistive technology in the new remote academic setting, and receive testing accommodations within the remote environment. While access and resources improved for students with disabilities over time, a portion of the surveyed disability resource professionals noted no observed improvement in students’ with disabilities communication with instructors as well as a worsening of conditions for students with disabilities throughout the pandemic related to access to counseling and mental health services. In addition to highlighting key obstacles faced by this student group during the pandemic, this paper provides recommendations and implications for institutions to better serve this student group, including how institutions of higher education can coordinate a holistic approach to support student mental health.

## 1. Introduction

Nineteen percent (19.4%) of U.S. undergraduate students and 11.9% of postbaccalaureate students enrolled in higher education report having a disability [1]. Although nearly one in five undergraduate students and one in ten postbaccalaureate students formally disclose their disability status, the actual number may be higher as students are not required to self-identify disability status to their institution’s disability resource office, may only share disability status with certain members of the campus community, or adjust disability status due to changing need for accommodations and student support services [2,3]. Students with disabilities in higher education are at a decreased chance to successfully access, persist, and obtain a postsecondary degree [4,5,6,7]. Students with disabilities have also shown delayed time to degree completion and decreased graduation rates [8,9]. The success of this student group is often challenged at various stages within their academic journeys and additional complications, including the impact of COVID-19, may further complicate their academic progress.

Since the beginning of the COVID-19 pandemic, higher education institutions have had to make significant adjustments to instructional delivery and administrative support to respond to required stay-at-home orders and safety protocols [10,11]. Emerging research indicates the educational experiences of students have been impacted due to the shift away from the “traditional” campus environment [12,13] with additional negative implications on specific at-risk student groups, including students with disabilities [14,15,16]. 

A significant number of students with disabilities in the higher education environment already face several challenges that may affect their overall success in the higher education environment, including decreased persistence and completion rates [17], as well as perceived stigma from members of the campus community [18,19]. The introduction of the COVID-19 pandemic brought on additional obstacles for students with disabilities, creating even more complications in their academic journey [20]. For example, Sutton (2020) found that during the pandemic, students with cognitive, learning, and physical disabilities felt less supported by the campus environment than peers without disabilities [21].

Research highlights that students with disabilities enrolled within the higher education environment have endured aggrandized barriers during the pandemic [22,23,24,25], with the experiences of specific disability groups, including mental health-related conditions, further exacerbated by the effects of COVID-19-related restrictions [26,27]. The rapid transition to remote instruction [28] and the fluctuating student support services available during the shift to online institutional environments [29,30], may have also negatively influenced students’ mental health related-conditions due to the disruption of daily routines, access to life necessities, and other associated factors [21,31]. Research from the Healthy Minds Network and American College Health Association found that students felt more stress due to thoughts about and/or possible experiences of exposure to COVID-19, financial stress related to the pandemic, and limited access to mental health care [32,33]. Emerging findings also show the significant number of young adults impacted by mental health problems related to life changes brought on by the COVID-19 pandemic [34,35,36]. Duquette (2000) found that creating community that supported and understood the needs of students with disabilities provided a greater opportunity to persist within the higher education environment [37]. However, the experiences of how students with disabilities are supported is often overlooked by higher education literature [38,39,40]. It is then critical to investigate how students with disabilities are supported and fare within the higher education setting, including during the COVID-19 pandemic. The aim of this paper was to explore disability resource professionals’ perceptions of access and overall student support of students with disabilities during the COVID-19 pandemic. Specifically, this study captured data at two unique time points—two months and nine months following the declaration of a worldwide pandemic. Utilizing data from disability resource professionals participating in a national survey initiative from the Association on Higher Education and Disability (AHEAD), this study focused on the perceptions of disability resource professionals on barriers to access and the potential change in student support for students with disabilities during the COVID-19 pandemic. In addition to highlighting the perceived critical obstacles faced by students with disabilities during the pandemic, this paper provides recommendations and implications for institutions to better serve students with disabilities and support disability support services within the higher education environment.

## 2. Methods

The data included in this paper were collected from a national survey exploring disability support services in higher education during the COVID-19 pandemic. As professionals who work directly with students with disabilities and guide campus response to barriers and access needs, these professionals provide a unique vantage point for understanding the impact of COVID-19 on students and the campus responses that are proving effective. This project investigated the perceptions and experiences of disability resource professionals supporting students with disabilities and managing disability support administrative work during the COVID-19 pandemic.

### 2.1. Recruitment and Participant Inclusion Criteria

Solicitation for survey participation occurred using the AHEAD membership roster. AHEAD is the largest national professional membership organization representing disability resource professionals and other campus constituents who work to assure equity for college students with disabilities. Individuals were considered eligible to participate in the project if they worked within a postsecondary institution’s disability resource office and AHEAD members. This paper includes data from two survey data collections—the first occurring in May 2020 and the second occurring in January 2021.

### 2.2. Sample

Disability resource professionals were the target administrator group for this study. Criterion sampling was used for this national project. This project accessed members of AHEAD—a professional membership association dedicated to promoting equity for individuals with disabilities in postsecondary education. Approximately 94% of AHEAD members work full-time in a disability resource office within institutions of higher education [41]. The first wave of data included 535 AHEAD members and the second wave of data included 631 AHEAD members. Participants ranged in their years of work experience, with 34.2% working 1–5 years and 14.0% working over 20 years in a postsecondary disability resource office. Three-quarters of participants (75.0%) possess a master’s degree. The large majority identified as female (77.3%) and White (84.3%). AHEAD members’ professional roles vary, including directors, coordinators, and specialists employed within higher education institutions’ disability resource offices. The institutional profiles of participants can be found in Table 1. 

### 2.3. Instrument and Analysis 

Data for this project were collected from a national survey of disability resource professionals. After institutional IRB approval was granted, the project solicitation initially occurred in May 2020 following AHEAD approval. This project was spearheaded by the authors and facilitated through AHEAD. The data included in this paper include survey responses from active AHEAD members and was extracted from the first two data collection periods—May 2020 and January 2021. The survey instrument was informed by the EDUCAUSE COVID-19 QuickPoll [42] and Biennial AHEAD Survey of Professionals [43]. The survey instrument also included questions on the transition to remote education, changes of operations for disability resource offices, and challenges and solutions identified throughout the pandemic. Data included within this paper were part of a larger national project exploring how disability resource professionals supported students with disabilities and campus accessibility throughout the COVID-19 pandemic [15,44]. More specifically, the first data collection focused on the initial pandemic-related challenges faced in access to technology, availability of student support services in the rapid shift from in-person to online modalities, and changes in institutional resources to support disability support services. The second data collection focused not only on initial pandemic-related challenges faced by disability resource professionals but also areas of continued obstacles, as well as areas of improvement nearly a year into the COVID-19 pandemic. The instrument was organized using an electronic survey platform. Prior to dissemination, the authors piloted the survey with disability resource professional content experts to ensure appropriate question development and accessibility of the instrument. The surveys were untimed, and all questions were made optional to complete. This project utilized descriptive statistics to analyze the national survey data.

## 3. Findings

Utilizing national survey data, findings included in this paper detail the academic and access challenges created by the pandemic for students with disabilities, as perceived by disability resource professionals. Data included in this paper capture disability support service challenges at two unique timepoints during the COVID-19 pandemic—two months [*n* = 535] and nine months [*n* = 631] following the worldwide pandemic designation starting in March 2020.

### 3.1. The Experiences and Perceptions of Disability Resource Professionals: Initial Lockdown

Two months into the pandemic, higher education institutions were still trying to grasp how to best support students safely and appropriately. By May 2020, the first data collection of this project, 99.4% of surveyed disability resource professionals noted their institutions had transitioned all coursework to a fully remote instructional format. When asked if their institutions had a clear plan for scheduled course delivery for the fall 2020 semester, 67.1% of surveyed disability resource professionals indicated that their institutions were still unsure of the confirmed fall 2020 semester instructional format. 

Although all students may have experienced some stressors related to the pandemic, data reveal that disability resource professionals perceived that students with disabilities may have faced additional obstacles during the transition to a fully online higher education environment. As noted in Table 2, based on their work and interactions with students with disabilities, surveyed disability resource professionals perceived overall difficulty for the student group as they transitioned to a fully remote educational experience. Specifically, survey participants reported that students with disabilities were having the greatest difficulty with Wi-Fi access (90.9%), possessing needed technology to support remote learning (82.6%), accessing to needed health services (82.2%), accessing to financial services (81.4%), and accessing to housing and food (80.2%). 

In addition to students with disabilities needing to adjust to the transition to remote coursework, the student group also needed to interact and work with their institutional disability resource office remotely. Surveyed disability resource professionals noted several perceived areas of difficulty for students with disabilities when working with their local disability resource office. As detailed in Table 3, surveyed participants perceived the greatest difficulty for students was if they needed to provide documentation for a disability (65.8%), use assistive technology in the new academic setting (62.1%), receive testing accommodations within the remote environment (61.9%), discuss new access barriers and solutions (60.9%), and receive notetaking accommodations (54.7%). 

### 3.2. The Experiences and Perceptions of Disability Resource Professionals: Nine Months into the Pandemic

Following the initial spring 2020 semester impacted by the pandemic, postsecondary institutions had the opportunity to reassess student needs and instructional formats for the fall 2020 semester. Beginning in the fall 2020 semester, only 8.2% of surveyed disability resource providers noted their institutions were entirely remote, with the majority of surveyed participants noting their institutions were utilizing a mix of both virtual and in-person learning opportunities (84.6%). However, as the fall 2020 semester progressed, and the second spike of the pandemic occurred, postsecondary institutions had to respond to the rising numbers of reported cases potentially impacting their campus community. Nearly half (44.0%) of survey participants noted their originally scheduled instructional format changed following the start of the fall 2020 semester. Of the participants who shared their institutions changed the instructional format following the start of the fall 2020, nearly all were from institutions who originally planned for a fully in-person or in-person/virtual mix (93.1%).

Following the completion of the spring and fall 2020 semesters, disability resource professionals began to reflect on the areas that were perceived as most challenging for students with disabilities during the transition to remote education. Although survey participants noted improvements in all areas, access to housing and food (27.4%) was the area of least improvement. Nearly one-quarter of survey participants also noted no observed improvement in students’ with disabilities communication with instructors (22.3%). Additionally, survey participants observed a worsening of conditions for students with disabilities throughout the pandemic related to access to counseling and mental health services (15.7%) and the communication and collaboration with other students (16.7%). A full list of perceived barriers for students with disabilities nine months into the pandemic can be found in Table 4.

As postsecondary institutions were forced to adapt to new procedures both in instructional and administrative support formats, students with disabilities were faced with changes in the procedures for identification and delivery of required accommodations. After the initial format changes occurring following the start of pandemic, administrators were required to modify traditional student support services to adhere to the remote postsecondary environment. This translated to modifications in the planning and use of disability accommodations within the remote learning environment. As noted in Table 5, survey participants perceived, overall, that the redevelopment and delivery of disability support services were effective in supporting students’ with disabilities requests and use of accommodations within the postsecondary environment. Nearly all survey participants perceived their disability resource offices were effectively providing student support for students with disabilities who requested previously approved accommodations (94.0%), discussed new barriers and solutions for updated accommodation plan use (93.5%), and participated in the interactive process of accommodation plan development (93.1%). However, obtaining note taking accommodations was perceived as the least effective area of support for students with disabilities during the transition and participation in the remote learning environment (9.9%). 

### 3.3. Changes to Accommodation Requests and Administrative Resources

In the spring 2020 semester, for students already enrolled with their institutions’ disability resource offices, 34.5% of surveyed disability resources professionals noted that their offices saw an increase in additional accommodation requests for previously registered students. Additionally, 40.2% of surveyed disability resource professionals noted an increase in new requests for students not previously registered with their institutions’ disability resource offices. 

Following the initial postsecondary response to the pandemic, disability resource offices reassessed student accommodation plans based on the changes in instructional formats. Forty-two percent (42.1%) of surveyed disability resource professionals indicated they noticed a documented increase in requests for accommodations and services during the fall 2020 term, when compared to the fall 2019 semester. Additionally, 46.0% of surveyed disability resource professionals observed a documented increase in registration and use of disability resource services in the fall 2020 term compared to the pre-pandemic fall 2019 semester. Survey participants also noted a significant increase in accommodation requests specifically related to students’ mental and emotional health during the fall 2020 semester. Over half of the wave 2 sample (59.4%) noted a documented increase in students registering with their institution’s disability resource office for mental health student support services.

With the documented increase in both accommodation requests for new students, as well as continued accommodation use for previously registered students with disabilities, continued institutional support is warranted to maintain the work and initiatives within disability resource offices. However, disability resource professionals noted significant financial constraints following the start of pandemic-related institutional budget cutbacks. Specifically, survey participants who replied to this question (*n* = 399) noted a reduction in their operating budget (63.3%), reduction of professional development funds (61.3%) and reduced access to resources necessary for administrative tasks (30.8%).

## 4. Discussion and Implications

Findings from this research work reveal the initial and ongoing challenges related to disability support services within the higher education environment during the first year of the COVID-19 pandemic. Data from disability resource professionals during the initial data collection occurring two months following the start of the COVID-19 pandemic revealed that the rapid shift to remote education complicated students’ with disabilities access to basic needs and necessary disability support services. Additionally, disability resource professionals noted the significant challenges occurring with their own professional obligations, creating obstacles with providing disability support services and operating their institution’s disability resource offices during the work-from-home restrictions. As time progressed, disability resource professionals noted that while institutions hoped to resume traditional learning and student support modalities, the second wave of the pandemic disrupted these plans, shifting many institutions back to a remote format. With time, improvements with perceived student access to basic needs and disability support services improvement; however, specific areas of students’ academic lives remained the same or worsened. Moreover, disability resource professionals noted the increase of requests and use of accommodations during the first year of the COVID-19 pandemic, when compared to pre-pandemic levels. The disability resource professionals who shared their perspectives and experiences in the AHEAD national surveys provided numerous accounts of how the rapid changes in operations across instructional and administrative support formats have both highlighted and at times exacerbated existing barriers for students with disabilities. Findings also revealed that as higher education institutions were forced to adapt to new procedures both in instructional and administrative support formats, students with disabilities were faced with changes in the procedures for identification and delivery of required accommodations. Their reports of what is working to ameliorate the barriers are instructive and inform the recommendations for higher education practices going forward. 

Before discussing the implications of this study, it is necessary to address the limitations of this work. As previously noted, this study captured self-reported data from disability resource professionals during the first year of the COVID-19 pandemic. It should be noted that any self-reported data can include potential flaws and misrepresentations. Additionally, while many questions focused on students with disabilities, ultimately only the perceptions of disability resource professionals on how students with disabilities fared were collected. The project would have improved if students with disabilities were directly surveyed on these questions. Additionally, surveying students without disabilities and comparing the two student groups would have also provided an important perspective as well. Survey data used in this study were collected in May 2020 and January 2021 and participants’ responses may have changed if data collection occurred at different points within the pandemic as the COVID-19 pandemic impacted individuals differently at different times. Moreover, response rates were not collected for this project. Lastly, the disability resource professionals included in this project were drawn from AHEAD membership. Although AHEAD possesses the largest national listing of disability resource professionals within the United States, it does not necessarily reflect all disability resource professionals working within U.S. higher education.

The observations and lessons learned from this large number of disability resource professionals throughout the higher education environment point to three broad shifts in thinking that are supporting student-centered responses during this time of continued change and evolution of college environments heading into a post-COVID world. These shifts in thinking are described below and followed by examples of approaches and strategies used on different campuses. The list of examples is not exhaustive and, given the wide diversity of institutions of higher education that are characterized by different missions, student populations, and resources, it is not expected that all strategies will be a fit for all campus environments. However, the examples illustrate innovative campus responses during the COVID era, providing a more intentional and holistic approach for including students with disabilities in the post-COVID higher education environment. 

### 4.1. Shift #1: Institution-Wide Planning and Systemic Approaches

The range of challenges experienced by students with disabilities during the pandemic—including areas such as technology, food and housing, financial aid, and mental health—clearly exceed the traditional focus of disability resource professionals on academic accommodations in the classroom. The role of electronic information technology in particular has been accentuated throughout the pandemic, and rapid changes in format have revealed barriers to broad educational systems including websites, learning management systems, and online instruction. Campuses that have responded with institution-wide strategies for identifying potential access barriers and addressing these inequities on a systemic level offer insight into approaches that not only assure access for students with disabilities but also benefit many students. Systemic solutions are sustainable over time and as illustrated in the examples below, in many cases reduce the need for retrofitted accommodations for individual students. What do disability resource professionals tell us is working?

With the abrupt changes to instruction and student services brought on by the pandemic in spring 2020, and continuing to impact higher education into 2021, campuses responded with a host of pandemic response teams, re-entry planning groups and ongoing task forces. The purpose of these structures was to gather campus leadership and thought to guide institutional response and action. Institutions that have tapped the disability expertise on campus for these ongoing discussions have benefitted from proactive conversations about barriers and possible implications for individuals with disabilities. 

Additionally, as campuses pass the one-year mark since COVID’s onset and plan for the new realities of higher education, many are reflecting on what pandemic responses will continue to benefit the campus. Campuses that implemented institution-wide strategies for the captioning of live and recorded video content are now well-positioned to meet legal requirements for online access that have been addressed in a piecemeal manner in the past. Many campuses described a process of better communication across campus offices assisting with captioning (e.g., the distance learning and disability resource offices), while others developed institutional contracts with outside captioning agencies. Additionally, institutions that embed accessibility tools within their campus learning management systems support faculty with the development and ongoing use of accessible online and hybrid classrooms. Institutional contracts for technology tools such as Blackboard Ally incorporate the process of reviewing online materials, identifying access barriers, and following tips to fix them directly into instructor course materials. Materials can more easily be converted into varied formats, such as PDF, audio, or ePub, a feature that can support not only students with disabilities but any student wishing to access materials in different ways. The Ally accessibility tool also enables campus-wide reporting so institutions can monitor progress in accessibility efforts. Lastly, recent experiences offering instruction in a variety of online and face-to-face formats is now being viewed on some campuses as an asset that can continue to allow students more choice and flexibility in course modalities in the post-COVID era. One approach described by a survey respondent for example was a campus plan to assure that courses that are offered in multiple sections include at least one on-line and one face-to-face delivery option. Continuing to incorporate strategies for flexibility and choice in course delivery make it easier for any student to choose the safest and most preferred course modality.

### 4.2. Shift #2: Cross-Campus Involvement and Collaboration

The restructuring of online instruction, student services, and campus events has expanded campus awareness of access issues. Campuses that have responded to the new awareness of barriers in the remote environment with enhanced training about inclusive strategies and increased collaboration in assuring accessibility for students with disabilities are discovering the value of a broadly informed campus community. The role of disability resource professionals in this environment has shifted to include enhanced campus outreach and training. Some campuses are recognizing the connections of disability inclusion with broader conversations of racial justice. Nearly all respondents reported increased collaboration and outreach with institutions’ teaching and learning centers, as well as instructional technology staff. 

As campuses become more aware of the scale of potential barriers to students with disabilities and more campus constituents recognize the value of their participation in ensuring inclusive environments, there is a need for training and information. Universal design is an approach to planning products and environments that anticipate human diversity and build in inclusive features. It has been successfully applied to a variety of contexts within higher education [45]. Disability resource professionals report a strategic use of existing online materials, free webinar opportunities, and local development of training materials to address the need for online information on topics ranging from disability resource office procedures to online accessibility, to inclusive teaching strategies. 

Additionally, there is perhaps no greater area of need for campus collaboration than the support of student mental health. In a timely study of barriers and supports for student mental health in remote learning settings, Lister, Seale, and Douce (2021) identified multiple sources that contribute to student wellbeing in the online environment and recommended that “universities need to tackle barriers where they reside, including exploring inclusive design practices for curricula and assessment, explicitly teaching study skills, designing systems and processes that do not cause undue stress and designing learning spaces to be communities” (p. 24) [45]. Disability resource professionals contribute to this cross-campus endeavor. While the community as whole works to provide a supportive environment for the many students who are experiencing increasing mental health concerns, some of these students will experience an impact on their mental health that reaches the threshold of being considered a disability and requires accommodation. Disability resource professionals have longstanding experience with the process of making this distinction with students with different kinds of non-visible disabilities including, for example, students with cognitive disabilities such as attention deficit disorders and learning disabilities, as well as students with more transient disabilities such as some forms of chronic health and psychological disabilities. To document the presence of a disability, a student must follow the institution’s procedures for documenting and substantiating a significant impairment. Disability resource professionals report a number of strategies to support and facilitate this process during the COVID transitions including, for example, developing secure online portals for submitting documentation, conducting intake interviews via Zoom, accepting tele-assessments conducted by qualified counselors or therapists, and providing early outreach to students during new student orientation. Importantly, disability resource professionals are also examining the impact of long-standing institutional requirements for disability documentation from a social justice perspective. Working to revise documentation requirements to emphasize more equitable approaches to establishing significant impairment in order to qualify for disability accommodations will be important work on the post-COVID campus. The guidance on documentation practices from AHEAD remains a useful resource in this process [46]. 

While disability resource professionals continue to support students, who qualify for disability accommodations, the proactive work of participating in campus planning and collaborating with campus colleagues to increase inclusive practices help to support all students on campus. Community awareness and use of inclusive practices is especially important for the many students experiencing mental health concerns who do not qualify as having a disability per se, but are experiencing hardship, nonetheless. Consider, for example, the accommodations that students with psychological disabilities frequently requested through the disability resource office: extended time on tests and flexibility with attendance requirements. When an instructor designs a course that builds in flexibility in these areas there is reduced need for formal accommodations through the disability resource office. 

### 4.3. Shift #3: Technology Enhanced Operations with Intentional Focus on Connections with Students

The perspectives and data from campus-based disability resource professionals over the course of the pandemic described a working environment where offices and staff members are being asked to do more with less. Disability resource offices have faced growing numbers of student accommodation needs, increasing numbers of students who register with the office, and increases in specific student populations including those with mental health, chronic health, and attention deficit disorders. Disability resource offices that had digitized student records and data management systems for implementing and monitoring student services prior to the pandemic were well positioned to make the shift to remote service provision. Many other disability resource offices however needed to make a significant transformation in service delivery. 

With the onset of remote services, disability resource professionals recognized that intentional effort would need to be placed into communicating with students in new ways. Disability resource offices have expanded the options for connecting with students. Providing multiple ways for students to get answers for questions, connect with staff, and receive services are helping to reach a variety of students with different communication and technology needs. Virtual additions such as adding a chat box on the office home page, providing virtual “walk in” hours to speak with staff, and using Zoom or Microsoft Teams for tutoring services are among the strategies offices are finding useful. For students who prefer in-person meetings reserving a large space for meeting that allows for social distancing is an option.

One aspect of assuring an equitable college experience for students with disabilities is to minimize the added burden required to request and use services [47]. Common strategies to promote ease of use among these new operational procedures included such approaches as creating a virtual “front desk” on the home page of the office website where students can directly access online appointment booking through features such as Microsoft Teams or Calendy. Student questions or concerns can be addressed immediately via a monitored chat function or expanded use of social media platforms. Procedures for required forms and disability documentation that are historically paper-intensive were converted to a more user-friendly documentation portal allowing for submission of secure online forms and provision of signatures through Docusign. Offices that acquired a data management system specific to disability resource office work such as AIM or Accommodate were able to support student ease of access through a seamless online request process where students can request Letters of Accommodation be directly e-mailed to faculty. Data management systems also provide much needed support for staff in their daily communication, tracking of service provision, and data collection.

## 5. Conclusions

As detailed in this paper, disability resource professionals perceived significant barriers for students with disability in their access to basic needs and disability support services during the COVID-19 pandemic. Additionally, disability resource professionals indicated their own professional challenges in supporting student needs through their institution’s disability resource office throughout the pandemic. Findings from the AHEAD national survey detail disability resource professionals’ experiences in supporting students with disabilities, as well as their participation in the necessary planning and implementation of strategies to best support institutional accessibility and accommodation plan development for student needs. Overall, findings revealed barriers in supporting disability-related needs throughout the higher education environment, including the increase in accommodation requests with the decrease of office budgets. Additionally, the disruption to the “typical” higher education environment contributed to the increase in students’ negative mental health. While the higher education community will feel the impact of the COVID-19 for some time, it is essential that administrators continue to consider and support the needs of students’ mental health, as well as identified disability statuses. 

## Figures and Tables

**Table 1:** Institutional profile of survey participants, by percent.

	Wave 1 (*n* = 535)	Wave 2 (*n* = 631)
Institution Type		
Private	36.3	34.1
Public	63.6	61.4
Other	0.2	1.7
Not Applicable	0.0	2.8
Minority Serving Institution (MSI)	23.2	17.0
Institution Size		
Less than 1500 Students	11.4	8.7
1500–9999 Students	47.1	41.7
10,000–19,999 Students	17.8	19.3
20,000–29,999 Students	9.9	10.2
More than 30,000 Students	13.8	20.1

**Table 2:** Disability resource professionals’ perceived barriers of students with disabilities during COVID-19 (two months into pandemic, *n* = 535), by percent.

	Difficulty
Access to network/Wi-Fi access	90.9
Having needed equipment/devices	82.6
Access to health services	82.2
Access to financial services	81.4
Access to housing and food	80.2
Communication/collaboration with other students	78.2
Communication with instructors	77.9
Access to needed technology support and/or training	77.3
Access to counseling/mental health services	69.7
Using the learning management system (e.g., Blackboard, Canvas, Moodle, etc.)	68.7
Access to software, assistive technology programs	68.5
Access to learning materials, course texts	64.2
Access to academic support/tutoring	63.6
Access to course assessments or exams	63.3
Access to library resources	60.7
Receiving previously approved accommodations	55.6
Access to academic advising	51.1

**Table 3:** Disability resource professionals’ perceived support service challenges for students with disabilities and disability resource office services during COVID-19 (two months into pandemic, *n* = 535), by percent.

	Difficulty
Providing documentation of a disability (if needed)	65.8
Using assistive technology	62.1
Receiving testing accommodations	61.9
Discussing new access barriers and solutions	60.9
Receiving notetaking accommodations	54.7
Participating in the interactive process	50.6
Receiving materials in alternate format	42.6
Receiving interpreter services	39.4
Requesting previously approved accommodations	39.1
Receiving CART/typewell services	39.0

**Table 4:** Disability resource professionals’ perceived barriers for students with disabilities during COVID-19 (nine months into pandemic, *n* = 631), by percent.

	Improvement	No Improvement	Worsening of Conditions	Never a Barrier	Not Sure
Needing Devices/Equipment	48.7	15.6	4.0	15.6	16.0
Access to Software, Assistive Technology Programs	45.7	17.1	4.0	20.4	12.8
Access to Network/WiFi	45.7	17.6	8.0	9.1	19.7
Access to Counseling/Mental Health Services	44.8	13.7	15.7	9.4	16.3
Communication with Instructors	36.1	22.3	14.3	11.3	16.0
Access to Housing and Food	27.4	13.9	14.9	14.1	29.7
Communication/Collaboration with Other Students	27.3	21.1	16.7	8.8	26.2

**Table 5:** Disability resource professionals’ perceived effectiveness of support service use for students with disabilities (nine months into pandemic, *n* = 631), by percent.

	Effective	Not Effective	Do Not Know
Requesting Previously Approved Accommodations	94.0	0.2	5.8
Discussing New Access Barriers and Solutions	93.5	0.4	6.1
Participating in Interactive Process	93.1	0.9	6.0
Providing Documentation of Disability	90.7	2.7	6.5
Receiving Testing Accommodations	89.9	2.2	8.0
Receiving Materials in Alternate Formats	88.2	1.3	10.5
Using Assistive Technology	85.9	2.9	11.2
Receiving Note Taking Accommodations	74.4	9.9	15.8
Receiving Interpreter Services	67.1	2.1	30.8
Receiving CART/Typewell Services	63.4	2.1	34.5

## Data Availability

The data presented in this study are available on request from the corresponding author. The data are not publicly available.
